# Individualised dose mapping uncertainty estimation in the reirradiation setting^☆^

**DOI:** 10.1016/j.phro.2025.100881

**Published:** 2025-12-02

**Authors:** Chelmis Muthoni Thiong’o, Marcel van Herk, Kathryn Banfill, Clara Chan, Catherine Harris, Matthew Lowe, Tom Marchant, Iskandar Mohamed, Golnoosh Motamedi-Ghahfarokhi, David Thomson, Ane Appelt, Eliana Vasquez Osorio

**Affiliations:** a Division of Cancer Sciences, Faculty of Biology, Medicine & Health, The University of Manchester, Manchester, UK; b Radiotherapy-Related-Research Group, The Christie Hospitals NHS Foundation Trust, Manchester, UK; c Department of Clinical Oncology, The Christie Hospitals NHS Foundation Trust, Manchester, UK; d Department of Medical Physics and Engineering, The Christie Hospitals NHS Foundation Trust, Manchester, UK; e Leeds Institute of Medical Research, University of Leeds, Leeds, UK; f Department of Medical Physics, Leeds Cancer Centre, Leeds Teaching Hospitals NHS Trust, Leeds, UK

**Keywords:** Reirradiation, Dose mapping uncertainties, Deformable registration, Head and neck cancer, Lung cancer

## Abstract

**Background and purpose:**

Deformable image registration (DIR) allows assessing radiation doses of previous treatment courses in reirradiation planning scans. However, DIR can introduce uncertainties in dose mapping. Estimating these uncertainties for individual patients remains a key challenge. We developed an individualised approach to estimate uncertainties in dose mapping using a commercially available treatment planning system.

**Materials and methods:**

For 54 patients who underwent reirradiation (27 head and neck, H&N, and 27 lung cases), we performed 16 DIRs per patient. We assessed each DIR geometrically using the mean distance to agreement (mDTA) between mapped contours and the corresponding structures of 30 to 34 organs at risk (OARs). We then estimated dose mapping uncertainties for structures where DIR resulted in plausible registrations, defined as mDTA ≤ 0.3 cm. We calculated the uncertainty of dose-volume-histogram estimates (mean and D0.1 cm^3^) and voxel-wise uncertainties using standard deviation (SD). We also tested the impact of using fewer registrations.

**Results:**

DIR resulted in plausible mappings for both patient cohorts’ OARs, ranging between 78.6 % and 88.2 %, varying between patients and OARs. Mean dose uncertainties ranged between 0 Gy and 0.43 Gy, while larger differences were observed for D0.1 cm3, between 0 Gy and 0.78 Gy. As expected, large voxel-wise uncertainties were in regions of steep dose gradients. Performance between registration sets was similar.

**Conclusion:**

We developed a method to estimate dose mapping uncertainties with integrated quality control for reirradiation. Differences between patients were observed, justifying the need for individualised DIR assessment. Few registrations were a pragmatic approach.

## Highlights

• Method gives patient-specific dose and geometry uncertainty in reirradiation.
• Local plausibility filter improves reliability of DIR in complex anatomy.
• Six registrations balance efficiency and accuracy for most cases.
• Voxel-wise maps reveal spatial dose uncertainty for clinical planning.
• Geometric plausibility and dose uncertainty don’t always correlate.

## Graphical abstract

**Figure:**
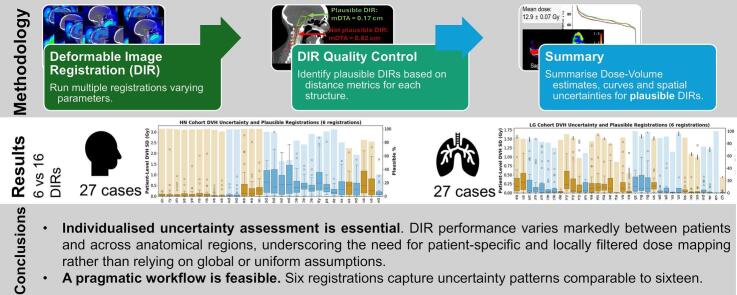

## 1 Introduction

Reirradiation is an increasingly viable option for patients with recurrent cancer, particularly in anatomically complex regions where surgery is not feasible [1,2]. Safe and effective reirradiation planning requires careful consideration of prior treatments, as cumulative doses can result in severe toxicities [1,3]. Ideally, reirradiation planning would be guided by the direct identification and quantification of radiation-induced tissue damage. However, this is not currently achievable using standard imaging or clinical biomarkers. Therefore, prior dose distributions are employed as practical, albeit imperfect, surrogates to inform reirradiation planning [4].

The European Society for Radiotherapy and Oncology − European Organisation for Research and Treatment of Cancer (ESTRO-EORTC) consensus on reirradiation recommends overlaying prior dose distributions when available, and dose accumulation in equieffective dose where feasible [5]. This requires aligning the anatomy of prior and current scans, often achieved through deformable image registration (DIR). DIR enables projection of prior dose distribution onto the updated anatomy, providing a voxel-wise estimate of cumulative dose [6,7].

However, the use of DIR in the clinical setting is hindered by uncertainties. Anatomical changes between treatment courses, e.g. (dis)appearance of anatomical structures, fibrosis, organ deformation and/or tumour (dis)appearance, can violate key assumptions of standard DIR algorithms. Such changes may be misinterpreted by DIR as motion or deformation, especially in contrast-devoid regions such as soft tissue in the head and neck, leading to uncertainties in dose maps [[6], [7], [8]].

In the absence of dose mapping, clinicians often assume a worst-case scenario where the highest dose points from each treatment course fully overlap spatially. While conservative, this assumption can severely restrict treatment options. By comparison, mapped dose distributions, despite uncertainties, can enable more tailored treatment plans [7,9].

In this study, we propose a novel method for individualised dose mapping uncertainty estimation in the reirradiation setting, aiming to provide physicians with additional confidence in the accumulated dose information, thereby supporting more informed, patient-specific treatment planning, while recognising that DIR can sometimes yield consistent but inaccurate mapping. We also investigate the performance of a pragmatic approach to our method. This is done on commercially available software for clinical applicability.

## 2 Materials and methods

### 2.1 Data

Radiotherapy data were available for 54 patients who underwent reirradiation [5] treated in a single institution. 27 patients were reirradiated for head and neck cancer (HNC) and 27 patients were treated for lung cancer (LC), see Table 1. Time between radiation courses ranged between 7 and 191 months, see Supplementary Table S1. We did not discriminate between new primaries and recurrences.

**Table 1:** Summary of the two patient cohorts, including the count of unique tumour sites and the mean and standard deviation of the interval, in months between the previous and reirradiation treatment courses. The count of unique tumour sites is the number of tumour sites in the entire cohort. For instance, the larynx, oral cavity, and oropharynx in the HNC cohort count as 3 unique tumour sites. See Supplementary Table S1 for details of individual patient tumour sites at both treatment courses.

Patient cohort	Count	Previous course count of unique tumour sites	Mean interval ± standard deviation (months)	Reirradiation course count of unique tumour sites
Head and neck cohort	27 patients	16 sites	41.8 ± 29.5	16 sites
Lung cohort	27 patients	5 sites	33.7 ± 36.4	8 sites

Planning computed tomographies (CTs), dose distributions and contours for the previous treatment as well as planning CT and contours for the reirradiation course, were imported into RayStation version 11B-R (RaySearch Lab, Stockholm, Sweden). These data were collected from the local research database (UK Computer-Aided Theragnostics (ukCAT) research database, ethics Research Ethics Committee reference number: 21/NW/0347, local consent reference 2022-017).

To ensure consistency in contouring and the same number of organs at risk (OARs) in both scans, we generated contours using the Limbus Contour deep-learning model (v 1.7.0-B3, Limbus.AI, Saskatchewan, Canada), resulting in up to 30 OARs for each patient in the HNC cohort and up to 34 OARs for each patient in the LC cohort.

### 2.2 Implementation

We implemented our tool to estimate individualised dose mapping uncertainties in a research version of a commercially available treatment planning system (TPS), RayStation (Version 11B-R, RaySearch Lab, Stockholm, Sweden), running on an NVidia Quadro RTX 6000 GPU, 6.4 GHz Intel(R) Xeon(R) CPU with 64 GB RAM. The method was run using RayStation’s scripting interface running CPython 3.8 (64-bit).

### 2.3 Registration

**Fig. 1:**
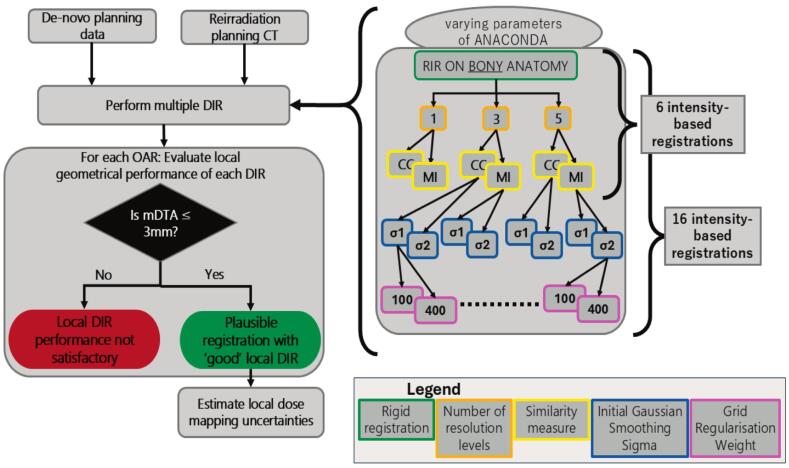
Flowchart of the script on RayStation that estimates dose mapping uncertainty for both **six** and **sixteen** registrations. Please note that we did not use one resolution level in the sixteen registrations. Supplementary Tables S2 and S3 detail the justification for our choice of parameters to vary. Note the registration quality control check that only maps the dose for OARs that are locally plausible. Abbreviations: CC − correlation coefficient; MI − mutual information.

Registration was performed between the previous and reirradiation planning CTs, using the reirradiation CT as the reference, see Fig. 1. We first did an intensity-based rigid frame of reference registration, focusing on aligning bony anatomy.

We then performed two sets of deformable registrations using one algorithm, the ANAtomically CONstrained Deformation Algorithm (ANACONDA) [9]. In the first set, we varied the similarity measure, either Correlation Coefficient (CC) or Mutual Information (MI) and the number of resolution levels, either 1, 3 or 5, resulting in six registrations. In the second set, we fixed the number of resolution levels at 3 and 5, the two similarity measures, but varied the grid regularisation weight (100 or 400) and the initial Gaussian smoothing sigma (1 and 2), resulting in 16 registrations.

We exemplified our approach using DIR configurations, selected based on prior literature [10]. Each DIR was performed for the complete CT scan with no OARs used as controlling or focus structures, and all other parameters set to their default values.

Registration performance was evaluated for all auto-segmented OARs using mean distance to agreement (mDTA) between the mapped structures and those defined on the reirradiation scan.

### 2.4 Dose mapping uncertainty estimation

DIR can perform differently across the scan, with regions where the alignment is acceptable or not [11]. We used this concept and the threshold proposed by AAPM TG-132 (mDTA ≤0.3 cm) to determine whether a registration was locally plausible [11].

To estimate dose uncertainty for a given OAR, we selected all locally plausible registrations to map the prior dose onto the reirradiation CT. We then calculated: 1) the mean and standard deviations (SD) of all plausible D0.1 cm^3^ and mean doses for serial and parallel organs, respectively, 2) DVH uncertainty, by plotting DVH curves of all plausible registrations, and 3) voxel-wise spatial uncertainty, by computing the SD of the mapped doses in all voxels within the OAR. Voxels were defined as those within the structure delineated on the reirradiation scan. D0.1 cm^3^ was defined as the dose to 0.1 cm^3^ of the OAR, representing a numerically stable estimate of the maximum dose, as used in our clinic for serial organs [12].

To determine general trends, we reported the number of plausible registrations across the complete patient cohort, discriminated by patient and OAR.

## 3 Results

It took around 15 min to run six registrations per single case, and 30–60 min to run 16 registrations per patient, with the lung cohort requiring more time due to the larger OAR volumes.

Overall, both sets of registrations performed better in the head and neck cohort, with 86.0 % and 88.2 % of the six and 16 registrations, respectively, considered plausible compared to 78.6 % and 82.8 % of the six and 16 registrations, respectively, in the lung cohort. Large variations were observed between patients in the number of registrations deemed plausible (Supplementary Figs. S5, S7, S9 and S10).

Using 16 registrations generally resulted in a higher percentage of plausible registrations than using six registrations (Supplementary Figs. S5, S7, S9 and S10). However, the geometric performance between the two sets of registrations followed similar patterns. E.g. patient L22′s registrations performed worst in the lung cohort (34.0 % and 44.2 % for six and 16 registrations, respectively, Supplementary Fig. S7).

Substantial differences were also observed in the registration performance per OAR, Supplementary Figs. S9 and S10. E.g. all registrations met the threshold for the cochleae in the HNC cohort. In the LC cohort, however, no OAR met the threshold across all patients. Registrations struggled particularly with the submandibular glands in the HNC and the anterior aorta base in the lung cohort.

**Fig. 2:**
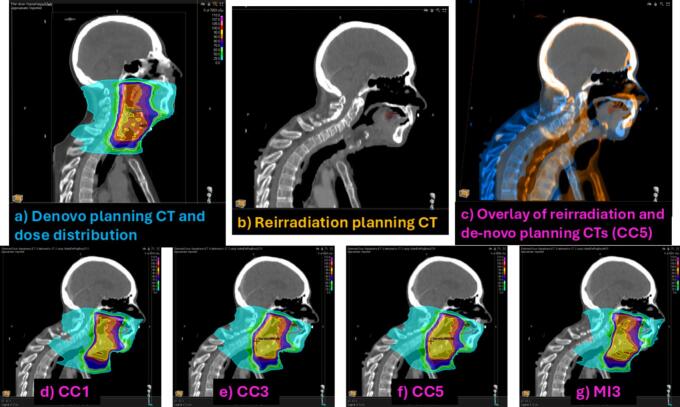
Patient H25 from the head and neck cohort, whose registrations performed worst in the head and neck cohort. a) The previous planning CT and dose distribution; b) The reirradiation planning CT; c) Overlay of the previous and reirradiation scans; d), e), f), and g) are the mapped dose distributions from some of the registrations performed, showing quite large variation. *CC − correlation Coefficient similarity measure, * MI − mutual information similarity measure, *1, 3, or 5 are the number of resolution levels. e.g. CC5 is a registration using correlation coefficient as the similarity measure and 5 resolution levels. Other examples are available in the Supplementary Figs. S1 to S3.

**Fig. 3:**
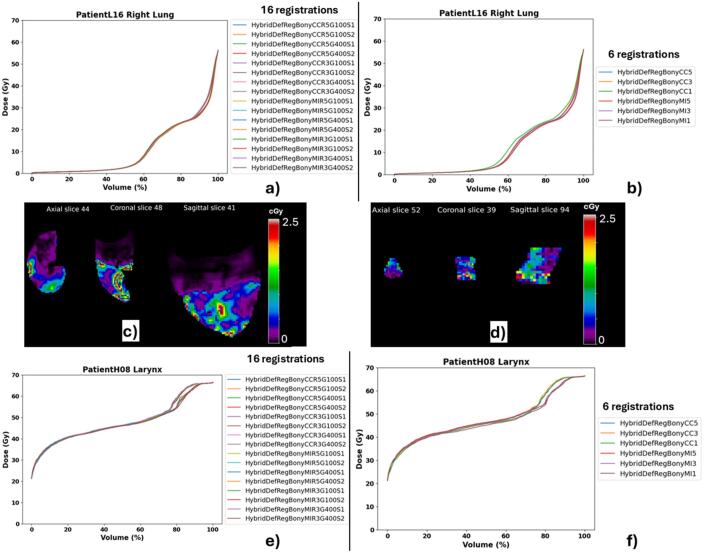
Dose mapping uncertainty estimation. Examples of DVH curves and spatial uncertainty maps for patient L16′s right lung (a, b, c), and H8′s larynx (d, e, f) are presented. In these cases, all registrations were deemed plausible. The DVH metrics for the selected OARs using 16 registrations: Right Lung: Mean dose = 10.4 ± 0.17 Gy, Larynx: Mean dose = 46.4 ± 0.3 Gy; and 6 registrations: Right Lung: Mean dose = 10.6 ± 0.45 Gy, Larynx: Mean dose = 46.1 ± 0.53 Gy. Within both OARs, the voxel-wise spatial uncertainty ranged from 0 to 0.25 Gy.

Fig. 2 shows an example of registration and dose mapping output. Fig. 3 shows examples of dose mapping uncertainty outputs for two OARs where all registrations were plausible (mDTA ≤0.3 cm). DVH plots were similar between the two registration sets for each OAR, indicating that a pragmatic approach (six registrations) provided similar information to 16 registrations. The voxel-wise spatial uncertainty maps (Fig. 3, Fig. 3) highlight the areas within the right lung and the larynx for patients H8 and L16, respectively.

**Fig. 4:**
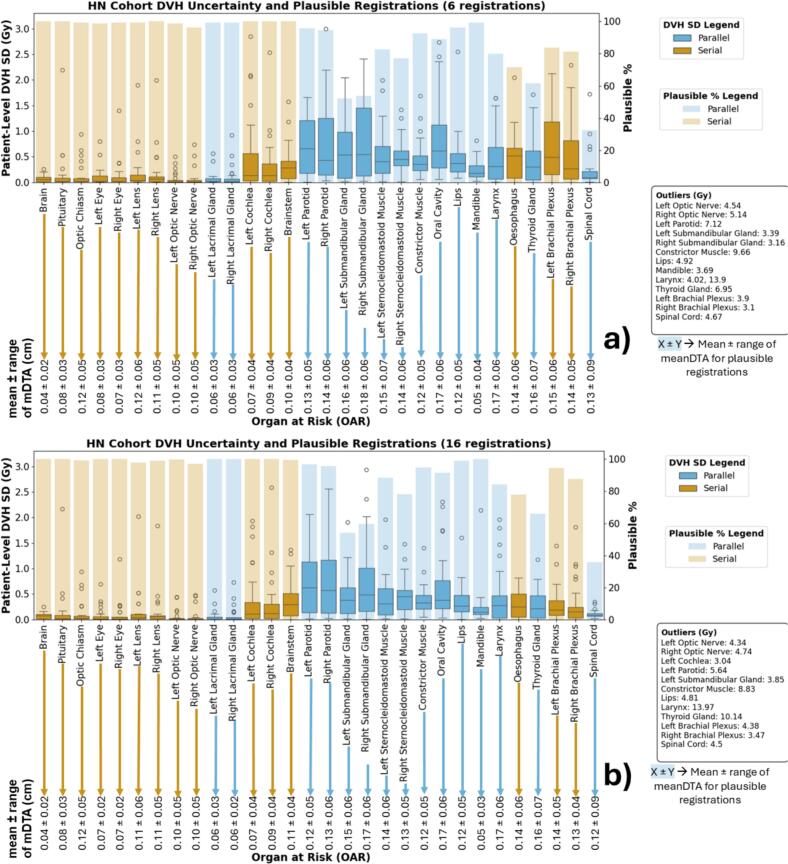
A summary of the DVH metric uncertainties for all OARs in the head and neck cohort using 6 (a) and 16 (b) registrations, organised by the general cranial-caudal format. For readability, outliers of each box plot are listed below the legends. The bar plots show the percentage of OARs that passed our set mDTA threshold of 0.3 cm (y-axis on the right-hand side of the plot). The box plots show the standard deviation of the mean dose for the parallel OARs and D0.1 cm^3^ for the serial OARs. The values at the bottom of each plot indicate the mean and range of mDTA values for the OARs whose registrations were plausible. A more detailed summary is available in Supplementary Tables S4 and S5. **Similar** trends in performance are observed between the two sets of registrations (see Supplementary Fig. S13).

**Fig. 5:**
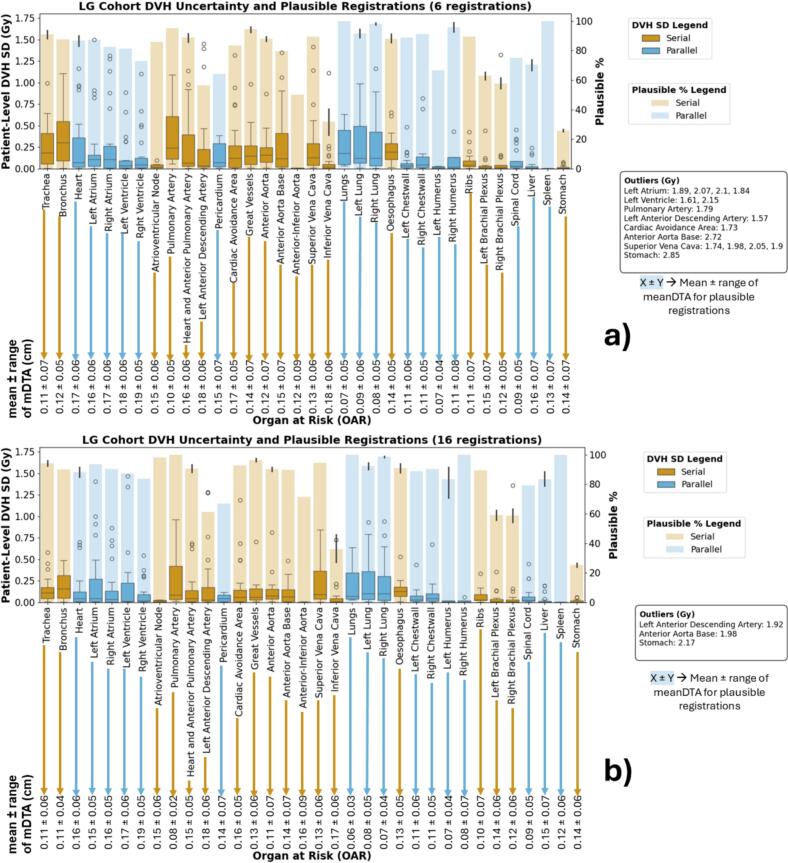
A summary of the DVH metric uncertainties for all OARs in the lung cohort using 6 (a) and 16 (b) registrations, organised by the general cranial-caudal format. For readability, outliers of each box plot are listed below the legends. The bar plots show the percentage of OARs that passed our set mDTA threshold of 0.3 cm (y-axis on the right-hand side of the plot). The box plots show the standard deviation of the mean dose for the parallel OARs and D0.1 cm^3^ for the serial OARs. The values at the bottom of each plot indicate the mean and range of mDTA values for the OARs whose registrations were plausible. A more detailed summary is available in Supplementary Tables S4 and S5. **Similar** trends in performance are observed between the two sets of registrations (see Supplementary Fig. S14).

Fig. 4, Fig. 5 illustrate overall uncertainty in each OAR across all patients, plotting the SD of the mean dose for parallel OARs and D0.1 cm^3^ for serial OARs. No clear correlation was observed in either cohort, even when OARs were grouped by type (serial vs parallel) or volume.

## 4 Discussion

We developed a method for individualised estimation of geometric and dose mapping uncertainties in the reirradiation setting. While previous work has explored uncertainty estimation by sampling across DIR algorithm parameters or using multiple algorithms, a key novelty of our approach lies in the additional filtering step to identify local plausibility. This accounts for the fact that DIR performance can vary significantly between anatomical regions of an individual patient, particularly in regions affected by anatomical changes such as tissue (dis)appearance.

Our results showed, indeed, that registration performance varied across patients and between anatomical locations within the same patient, with poor performance indicating that some patients’ anatomical changes were more challenging than others. While it is well-recognised that DIR algorithms’ performance varies, our findings extend this understanding to the reirradiation context. Using a single uniform uncertainty metric, such as fixed spherical margins [13], risks oversimplifying this patient-specific and spatial variability.

By performing multiple registrations and assessing geometric agreement, we provide a practical methodology for identifying unreliable dose mappings that highlight OARs requiring further scrutiny in clinical decision-making. This is particularly advantageous in reirradiation, where atypical anatomy is common, and standard deformable registration algorithms may not achieve reliable mapping across the complete image [6,9,13,14].

Many algorithms are purely intensity-based and do not incorporate controlling structures [[15], [16], [17], [18]]. It may be advantageous to use an algorithm that includes controlling structures, such as is available in RayStation. For example, Supplementary Fig. S4 shows a registration using controlling structures for patient H25, whose intensity-based registrations performed worst in the HNC cohort. The overlay demonstrates better alignment, illustrating that plausible alignment can be achieved by incorporating improved registration parameters or algorithms. However, it should be noted that controlling structures would not be used for evaluation because of overfitting.

Recent studies have proposed methods to incorporate uncertainty in dose mapping by applying worst-case scenarios across all mapped doses. For example, Garcia et al. applied an upper uncertainty bound derived from all registrations, while Thompson et al. and Mechalakos et al. used local maximum doses as conservative surrogates, and Meyer et al. used maximum doses within a neighbourhood [9,13,[19], [20], [21]]. In contrast, our method incorporated a local plausibility filter, enabling spatially resolved uncertainty quantification. This provides a more concise and locally relevant assessment of confidence in the mapped dose, which supports more informed treatment planning and decision-making. Similar approaches exist in the literature outside reirradiation [22,23]. For example, Hub et al. used the sum-of-square-differences metric, while Wang et al. applied a 0.3 cm distance criterion for 99 % of voxels. Importantly, none included voxel-wise spatial uncertainty estimation. Voxel-level uncertainty has the potential to influence reirradiation planning in regions where certainty is crucial, e.g. the base of the brainstem, associated with dysphagia [24,25]. STRIDeR (Support Tool for Re-Irradiation Decisions guided by Radiobiology), a pathway developed by Murray et al. in RayStation, also performs voxel-wise optimisation for high-dose recurrences [26].

Current approaches for estimating image registration performance rely on surrogate metrics such as contour overlap measures, which assess the geometric alignment between deformed and reference structures. However, these methods do not directly assess the impact of registration uncertainty on the mapped dose, limiting their clinical applicability [6]. Several techniques, including spherical or ellipsoid searches, have been reported [9], [13], [13], [20], but these approaches assume a constant level of uncertainty throughout the entire image.

We have demonstrated that DIR plausibility can differ within a single patient, reflecting challenges in regions with atypical anatomy where accurate structure-to-structure mapping is inherently difficult [13], [19]. García-Alvarez proposed a voxel-wise spatial uncertainty map using inverse consistency error (ICE) magnitude, which can be overridden or predefined, and adjusts for dose gradients [19]. A key difference in our approach is the use of the contour overlap measure, mDTA, to determine local plausibility, providing a pragmatic way to filter out registrations that would inaccurately map doses. By including only plausible registrations, our quantified uncertainty is meaningful and corresponds to Type A uncertainty estimation [11], [26], [27].

DIR plausibility assessment relied on deep-learning-generated contours. We used these instead of clinical contours, as the latter are subject to significant inter-observer variability (IOV) and evolving contouring practices. Moreover, clinical structure sets are not consistent between patients, which severely restricts their use in registration assessments. Modern auto-segmentation tools provide consistent OAR definitions and reduce variability [28], offering a more reproducible basis for evaluating DIR performance. While ideally auto-segmented contours should be reviewed and corrected by specialists, we performed spot-checks in our dataset and consider the current approach appropriate for this feasibility cohort study. Moreover, no impact has been reported in auto-segmentation in reirradiation anatomies [29]. A future application of our approach requires assessing the correctness and consistency of contours between scans.

Using 16 registrations modestly increased the proportion of plausible results compared to six, particularly in the lung cohort, but more than doubled the computation time. Geometric performance patterns, DVH and voxel-wise uncertainty maps showed comparable results, with six registrations. These findings suggest that six well-chosen registrations may offer a favourable trade-off between accuracy and efficiency, with additional configurations reserved for anatomically complex cases. Further work involving varying parameters or incorporating other registration algorithms to extend the variability of results in the uncertainty sources and external validation is underway.

The use of a 0.3 cm mDTA threshold was informed by AAPM TG-132 guidance; however, we recognise that recommendations suggest selecting thresholds relative to the voxel size. In our dataset, the in-plane voxel size was ∼0.08 cm, and the slice thickness was 0.25 cm, placing the 0.3 cm threshold within a reasonable range. Furthermore, organ-specific inter-observer variability (IOV) ranges, reported in studies such as Brouwer et al. and Vinod et al., highlight the potential of tailoring plausibility thresholds to individual structures, a direction we identify for future refinement of our framework [30], [31].

The time it took to generate the dose mapping uncertainty results was generally acceptable, but better streamlining would help improve acceptability in clinical settings. There are no studies that report the time it took to perform dose mapping uncertainty estimation specifically, though work by Mechalakos et al. reports time for steps in their dose accumulation with a built-in uncertainty computation script [9]. Studies have mentioned the time it took for a reirradiation plan generation, including the Special Medical Physics Consult Process (SMPC) for Reirradiation, which includes other considerations beyond image registration [13], [32]. Our aim was not to demonstrate the superiority of our method, but rather to showcase a quantitative patient-specific method for dose mapping uncertainty estimation in an automated manner.

This work aimed not to demonstrate which algorithms perform better than others, but to show that a patient-specific approach is crucial and to illustrate a method that can detect which patients or OARs have complex or atypical changes between treatment courses. To further investigate whether geometric plausibility is reflected in the variability of accumulated dose, we compared DVH standard deviation against registration plausibility (mDTA ≤0.3 cm). We found no consistent correlation, even when OARs were grouped by volume or functional type (serial vs. parallel) (see Fig. 4, Fig. 5). This demonstrates that geometric plausibility alone may not adequately capture dose mapping uncertainty across patients or OARs.

Ideally, reirradiation planning would be guided by direct identification of radiation-induced damage in the current anatomy. As no validated biomarkers currently exist, prior dose distributions mapped using DIR remain the most practical method for dose accumulation. A recent review by Beddok et al highlights the potential of radiomics and quantitative imaging biomarkers to inform reirradiation planning, but emphasises that their clinical adoption is limited by insufficient external validation, methodological variability, and insufficient evidence [33]. In recognition of these limitations, our approach incorporates uncertainty estimation to complement DIR mapped doses and support more robust interpretation.

The uncertainty maps produced in this study have several intended clinical applications, including supporting reirradiation planning and aiding decision-making during multidisciplinary team (MDT) discussions. However, how these uncertainties should be interpreted and acted upon remains case-specific. For example, large uncertainty in regions receiving negligible new dose may be clinically irrelevant, while small uncertainty near dose-limiting structures, particularly those close to tolerance limits, can directly influence treatment feasibility. This highlights the importance of presenting spatial uncertainty information alongside mapped doses to support clinical interpretation and nuanced decision-making. While DVHs offer a useful summary of dose distribution, they inherently lose spatial resolution and may mask localised uncertainties. Spatial uncertainty maps, by contrast, provide voxel-level insight, allowing clinicians to identify areas where dose estimates are more or less reliable. Intuitive reporting of these uncertainties remains an unresolved challenge. Although traffic-light systems have been proposed in other contexts [34], their suitability for dose mapping requires further investigation. Importantly, our method quantifies uncertainty but is not a detector of registration failure; low-uncertainty regions should still be interpreted with caution and clinical judgment.

The current implementation also allows for batch processing of large cohorts, which would enable outcome-modelling studies of reirradiation cases incorporating dose uncertainties. Registration using the open-source tool NiftyReg showed unsatisfactory geometric performance (Figure S16) compared to ANACONDA-based, clinically tuned methods for our patient cohort, suggesting that such research tools are not optimised for intra-patient radiotherapy registration. Future work will focus on optimising visualisation techniques and integrating uncertainty metrics into clinical workflows to enhance practical utility, with direct execution within RayStation’s scripting interface enabling clinical use.

In conclusion, our results highlight the variation in DIR performance between patients and OARs and their impact on dose mapping. Having a method to estimate uncertainties has been identified as a key gap in tools enabling reirradiation in the clinic [35], [36]. Estimating the uncertainties is the first step towards incorporating them both for multidisciplinary team discussions of potential treatment for new or recurrent tumours, as well as for optimising reirradiation planning. Ultimately, our goal is not to establish the superiority of this method over existing approaches but to provide a robust, individualised uncertainty estimation method that incorporates a quality control mechanism, thus enhancing clinical decision-making.

## CRediT authorship contribution statement

**Chelmis Muthoni Thiong’o:** Methodology, Software, Formal analysis, Data curation, Investigation, Visualization, Writing – original draft, Writing – review & editing. **Marcel van Herk:** Conceptualization, Resources, Writing – review & editing, Supervision, Funding acquisition. **Kathryn Banfill:** Data curation, Resources. **Clara Chan:** Data curation, Resources. **Catherine Harris:** Data curation, Resources. **Matthew Lowe:** Supervision, Writing – review & editing. **Tom Marchant:** Data curation, Resources. **Iskandar Mohamed:** Data curation, Writing – review & editing. **Golnoosh Motamedi-Ghahfarokhi:** Data curation, Writing – review & editing. **David Thomson:** Supervision. **Ane Appelt:** Supervision, Writing – review & editing. **Eliana Vasquez Osorio:** Conceptualization, Funding acquisition, Methodology, Writing – review & editing, Project administration, Resources, Supervision, Data curation.

## Declaration of competing interest

The authors declare the following financial interests/personal relationships which may be considered as potential competing interests: The authors declare the following financial interests/personal relationships which may be considered as potential competing interests:Chelmis Muthoni Thiong’o: None. Marcel van Herk: None. Kathryn Banfill: None. Clara Chan: None. Catherine Harris: None. Matthew Lowe: None. Tom Marchant: None. Iskandar Mohamed: None. Golnoosh Motamedi-Ghahfarokhi: None. David Thomson: None. Ane Appelt: Leeds Teaching Hospitals NHS Trust holds an institutional research collaboration agreement with RaySearch Laboratories, which includes reirradiation research & development. Eliana Vasquez Osorio: Guest Editor.
