# Supplementary material for: Individualised dose mapping uncertainty estimation in the reirradiation setting
Source: Phys Imaging Radiat Oncol. 2025 Dec 2;36:100881. doi: 10.1016/j.phro.2025.100881 (PMC12732311; doi:10.1016/j.phro.2025.100881)
Supplement: Supplementary Data 1 [file mmc1.pdf]

Supplementary Material

Patient Data Summary

Table S1: Summary of patient tumour sites, interval in months between the previous and reirradiation treatment courses and the reirradiation type for each patient.

Patient	Previous disease site	De-novo RT dose (Gy) / fractionation	Time interval to reirradiation (months)	Site of recurrence
H1	Left parotid	60 / 30	12	Paranasal sinus
H2	Right maxilla	63 / 30	65	Right maxilla
H3	Oral cavity	60 / 30	15	Left cervical nodes
H4	Larynx	66 / 30	65	Larynx
H5	Left tonsil	66 / 30	112	Hypopharynx
H6	Oral cavity	60 / 30	59	Hypopharynx
H7	Supraglottis	55 / 20	77	Hypopharynx
H8	Base of tongue	66 / 30	27	Oropharynx
H9	Oropharynx	63 / 30	70	Larynx
H10	Right neck	55 / 20	8	Left neck
H11	Nose	66 / 30	17	Right maxilla sinus
H12	Left sinonasal	60 / 30	33	Bilateral neck
H13	Oropharynx	66 / 30	28	Tonsil
H14	Right parotid	24 / 12	68	Right mandible
H15	Right maxilla	60 / 30	7	Neck nodes
H16	Left tonsil	60 / 30	12	Right neck
H17	Base of tongue	66 / 30	37	Right neck
H18	Nasopharynx	70 / 33	25	Left paravert
H19	Oropharynx	55 / 20	54	Oral cavity
H20	Nasopharynx	70 / 33	46	Oral cavity
H21	Hypopharynx	66 / 30	22	Left neck
H22	Larynx	66 / 30	19	Larynx
H23	Larynx	55 / 20	23	Larynx
H24	Buccal mucosa	60 / 30	79	Right neck

H25	Hypopharynx	66 / 30	103	Oral cavity
H26	Hypopharynx	66 / 30	27	Post cricoid
H27	Oral cavity	60 / 30	18	Right neck
L1	Left lung	55 / 20	12	Left lung
L2	Left lung	55 / 20	13	Right lung
L3	Right lung	55 / 20	58	Right lung
L4	Right lung	55 / 20	61	Left lung
L5	Right lung	55 / 20	13	Mediastinum
L6	Right lung	50 / 20	47	Left lung
L7	Right lung	66 / 33	62	Right supraclavicular fossa (SCF)
L8	Right lung	55 / 20	45	Right lung SABR
L9	Right lung SABR	60 / 8	13	Mediastinum
L10	Left lung	55 / 20	63	Left lung
L11	Right lung	48 / 3	15	Right lung
L12	Left lung	55 / 20	18	Left lung
L13	Left lung SABR	50 / 8	14	Left lung
L14	Right lung	66 / 33	191	Right lung
L15	Right lung	55 / 20	24	Left lung
L16	Right mediastinum	55 / 20	20	Right lung
L17	Left lung	55 / 20	7	Right lung SABR
L18	Left lung	45 / 30	49	Bilat lungs
L19	Right lung	60 / 5	15	Right lung
L20	Right lung SABR	50 / 8	18	Right lung
L21	Right lung	55 / 20	10	Right lung apex
L22	Right lung	55 / 20	37	Right lung
L23	Left lung SABR	60 / 8	11	Right lung
L24	Oesophagus	50 / 16	22	Right lung
L25	Bilateral lung SABR	60 / 5	33	Right lung
L26	Left lung	55 / 20	10	Upper left lung
L27	Right lung	55 / 20	28	Right upper mediastinum

Justification for DIR parameters

There are 2 algorithms in RayStation (Version 11 B-R, RaySearch Lab, Stockholm, Sweden): MORFEUS and ANACONDA. For our dataset (CT-to-CT registration), ANACONDA is appropriate based on the literature [1]. The available parameters within ANACONDA with their respective default values are shown in Table S2 below.

Table S2: Default parameters within the ANACONDA algorithm.

```
AlgorithmSettings={'NumberOfResolutionLevels': numResolutionLevels, 'InitialResolution': {'x': 1, 'y': 1, 'z': 1}, 'FinalResolution': {'x': 0.25, 'y': 0.25, 'z': 0.3}, 'InitialGaussianSmoothingSigma': 2, 'FinalGaussianSmoothingSigma': 0.333333333333333, 'InitialGridRegularizationWeight': 400, 'FinalGridRegularizationWeight': 400, 'ControllingRoiWeight': 0.5, 'ControllingPoiWeight': 0.1, 'MaxNumberOfIterationsPerResolutionLevel': 1000, 'ImageSimilarityMeasure': CorrelationCoefficient, 'DeformationStrategy': "Default", 'ConvergenceTolerance': 1E-05 }
```

Parameters were varied, resulting in 16 deformable registrations:

- Similarity Measures (2 values):
  - Correlation Coefficient
  - Mutual Information
- Resolution Levels (2 values):
  - 5 and 3
- Grid Regularisation Weight (2 values):
  - 100 and 400
- Initial Gaussian Smoothing Sigma (2 values):
  - 1 and 2

Parameters were varied, resulting in 6 deformable registrations:

- Similarity Measures (2 values):
  - Correlation Coefficient
  - Mutual Information
- Resolution Levels (3 values):
  - 5, 3 and 1

Table S3 shows justification for the choice of parameter variations.

Table S3: Justification for choice of parameter variations.

Parameter	Values varied	Clinical relevance in reirradiation (H&N, Lung)
**Varied parameters**		
Image similarity measure	Correlation Coefficient (CC), Mutual Information (MI)	CC: suitable when CT intensity relationships are consistent. It is essential for CT-to-CT registration across treatment courses.<br>MI: robust to intensity changes due to IV contrast, fibrosis, or anatomical distortion, which may be common in reRT planning from heterogeneous CT sources. MI improved accuracy in H&N CT-to-CT registrations and dose accumulation (±2.5 mm landmark error; ±0.9 Gy dose uncertainty)

		[2,3]
Resolution levels	5, 3, 1	Studies support multi-resolution FFD in hybrid DIR for H&N adaptive therapy (3 levels widely used). Multi-resolution hierarchy improves convergence:<br>• Higher levels (5) capture large anatomical shifts, e.g. weight loss, tumour shrinkage, post-surgical rearrangement.<br>• Lower levels (3) refine local accuracy around OARs and tumour. Critical when mapping prior and new planning CTs with gross changes.<br>[2,4]
Grid regularisation weight	100, 400	Controls the smoothness of the deformation field, thereby adjusting deformation flexibility. Lower weight permits soft-tissue warping (e.g. parotids, lung apex); higher weight avoids overfitting in bony regions or low contrast zones. A review on DIR uncertainties emphasises the need to tune regularisation per anatomical site. Consistency between initial and final stages prevents unexpected stiffness changes mid-registration, ensuring predictable deformation.<br>• High weight (400): yields smoother, more anatomically plausible deformations, important for rigid structures like airway, bone.<br>• Low weight (100): allows more local deformation, better for regions with soft-tissue change (parotids, lungs). Balances under- vs over-regularisation.<br>[5,6]
Initial Gaussian smoothing sigma	1, 2	Literature identifies smoothing as a key regularisation control affecting DIR precision in adaptive RT. Higher smoothing increases robustness to noise/artefacts but may blur small features, thereby influencing registration sensitivity to subtle differences between CTs; lower smoothing retains structural detail.<br>Important trade-off in post-treatment CTs with surgical or contrast variability.<br>[4,5,7,8]

Registration Results (Visual Inspection)

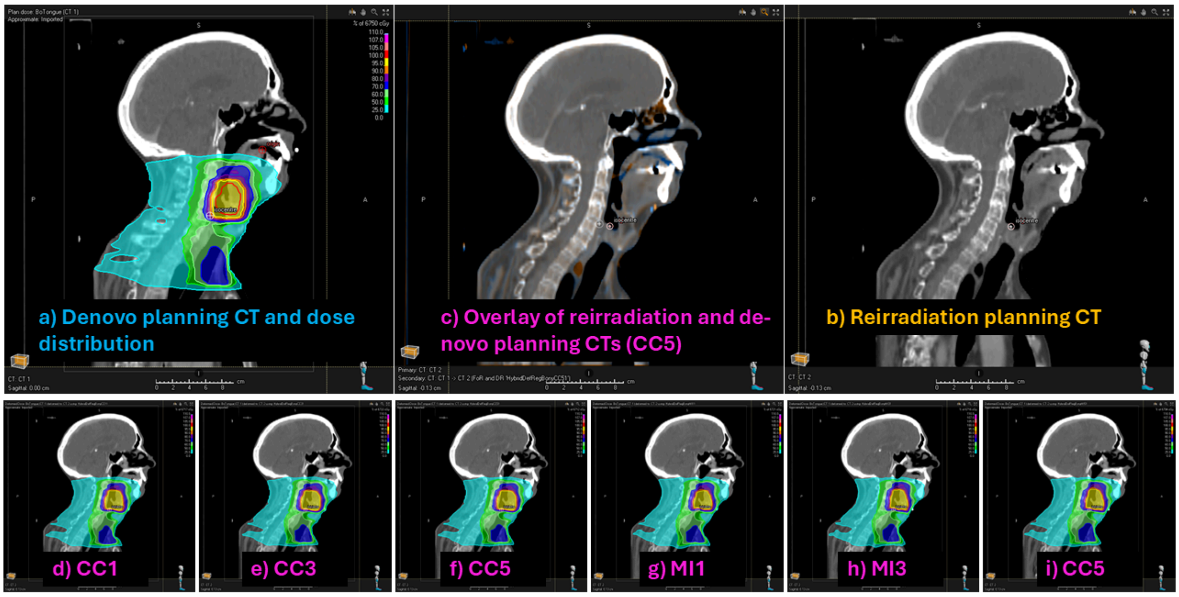

Figure S1: Patient H8 from the head and neck cohort, whose registrations performed best in the head and neck cohort. a) is the previous planning CT and dose distribution; b) is the reirradiation planning CT; c) is the overlay of the previous and reirradiation scans; d), e), f), g), h), and i) are the mapped dose distributions from each of the 6 registrations performed. * CC - correlation Coefficient similarity measure,* MI - mutual information similarity measure. * 1, 3, or 5 is the number of resolution levels. e.g., CC5 is a registration using correlation coefficient as the similarity measure and 5 resolution levels.

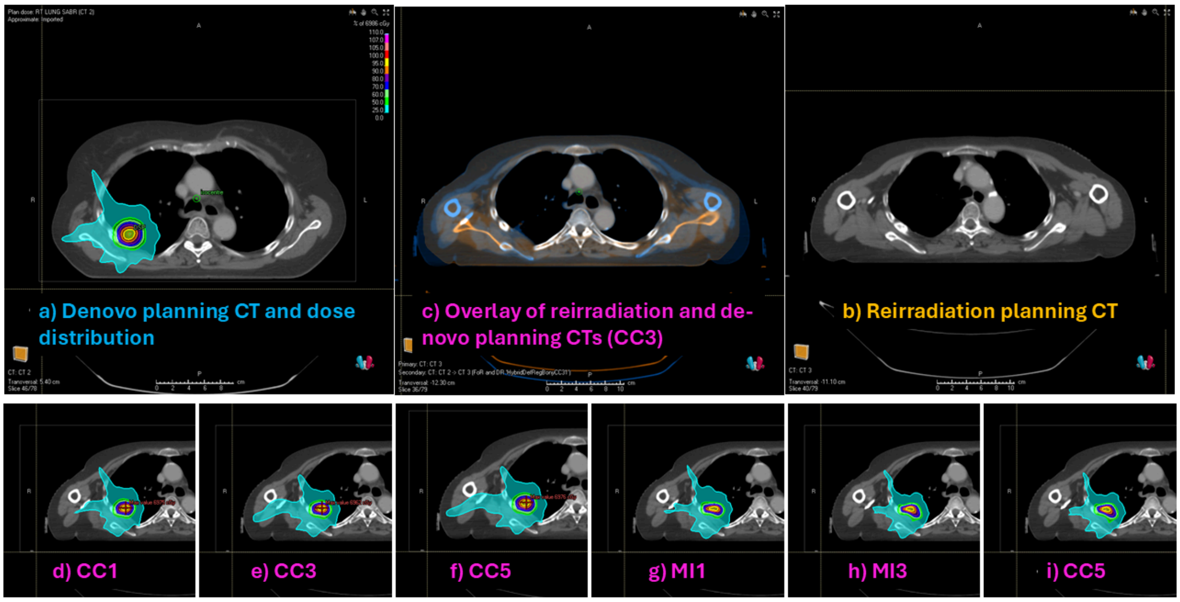

Figure S2: Patient L3 from the lung cohort, whose registrations performed best in the lung cohort. a) is the previous planning CT and dose distribution; b) is the reirradiation planning CT; c) is the overlay of the previous and reirradiation scans; d), e), f), g), h), and i) are the mapped dose distributions from each of the 6 registrations performed. * CC - correlation Coefficient similarity measure, * MI - mutual information similarity measure. * 1, 3, or 5 is the number of resolution levels. e.g., CC3 is a registration using correlation coefficient as the similarity measure and 3 resolution levels.

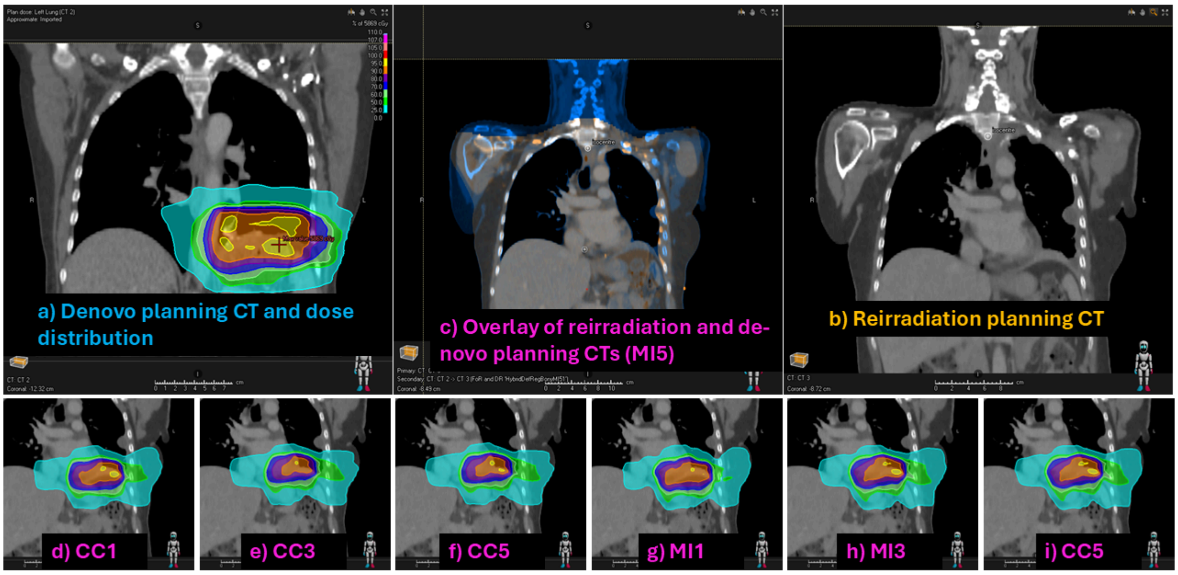

Figure S3: Patient L22 from the lung cohort, whose registrations struggled most in the lung cohort. a) is the previous planning CT and dose distribution; b) is the reirradiation planning CT; c) is the overlay of the previous and reirradiation scans; d), e), f), g), h), and i) are the mapped dose distributions from each of the 6 registrations performed. Of keen interest is that this patient had **different arm positioning** in the 2 treatment courses (arms-up in the previous scan, arms-down in the reirradiation scan). * CC - correlation Coefficient similarity measure,* MI - mutual information similarity measure. * 1, 3, or 5 is the number of resolution levels. e.g., MI5 is a registration using mutual information as the similarity measure and 5 resolution levels.

Registration with controlling ROI for the challenging case

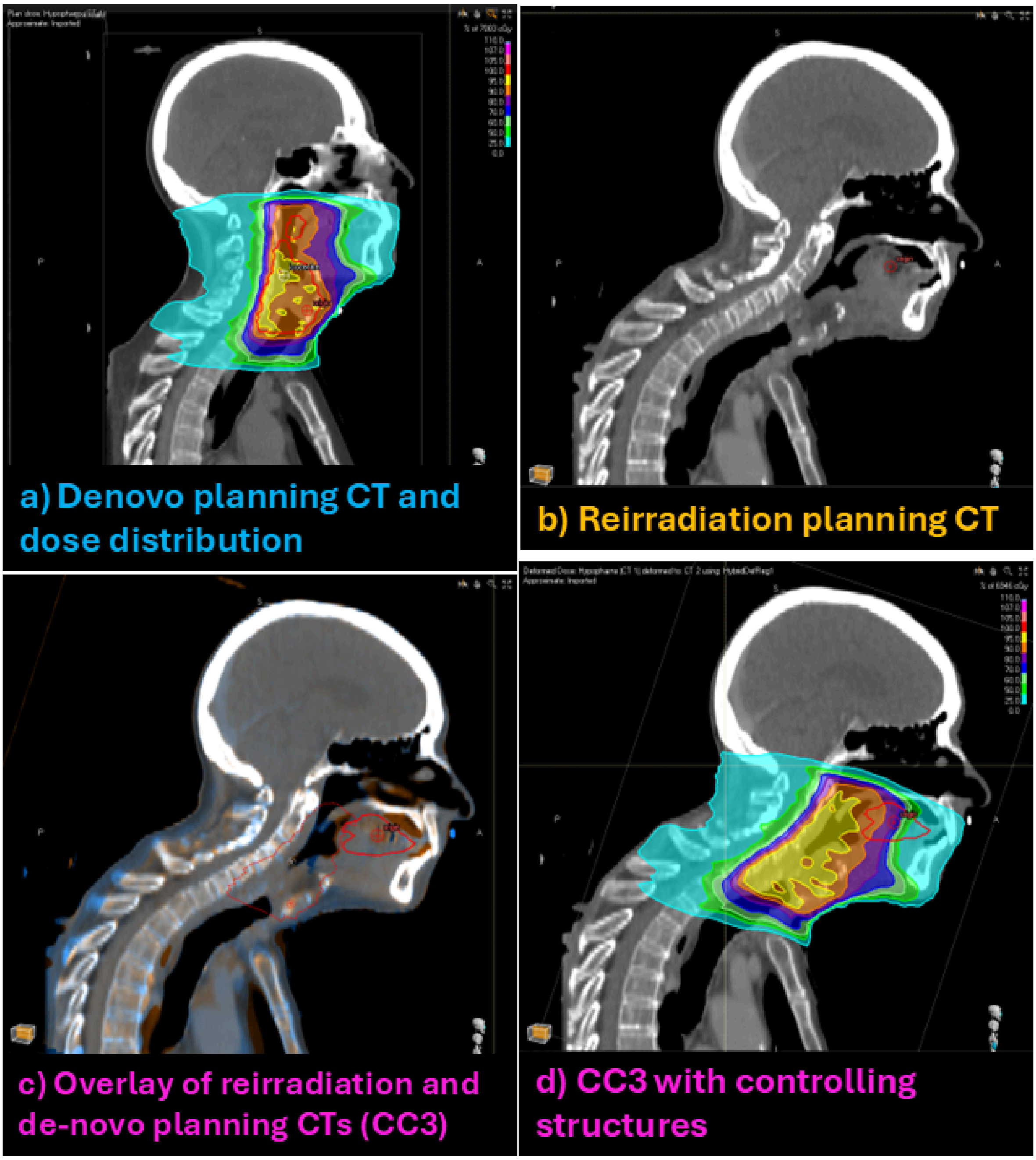

Figure S4: Patient H25 with the brainstem and spinal cord as controlling structures in the deformable registration. The resulting overlay of images c) is a dramatic improvement from Figure 3c), and this is also reflected in the resulting mapped dose d), which has not been mapped outside the anatomy as in Figure 3. This demonstrates that a change in algorithm parameters can lead to better registrations for patients whose registrations struggle.

Results on Plausible Registrations

Table S4: A summary of the DVH metric uncertainties for all OARs in our 2 patient cohorts using 6 registrations, including the count of OARs that passed our set mDTA threshold. The statistics are for the OARs whose registrations were plausible. Please note that the total count of the OARs considered varied in some cases, if the OAR was within the target volume or due to failure in the auto-segmentation. Some OAR names are duplicates due to instances of partial OAR being clinically delineated. These results were not merged with whole organ results, such as the stomach in the lung cohort.

OAR	Number of Plausible Registrations out of the total count of OARs considered	Mean ± Standard Deviation of mDTA (cm)	Mean ± Standard Deviation of mDTA (cm) for Plausible Registrations
Mandible	161 out of 162	0.05 ± 0.05	0.05 ± 0.04
Left Brachial Plexus	129 out of 154	0.21 ± 0.20	0.15 ± 0.06
Right Brachial Plexus	125 out of 154	0.24 ± 0.24	0.14 ± 0.05
Brain	162 out of 162	0.04 ± 0.02	0.04 ± 0.02
Brainstem	162 out of 162	0.10 ± 0.04	0.10 ± 0.04
Oral Cavity	144 out of 162	0.20 ± 0.09	0.17 ± 0.06
Left Cochlea	162 out of 162	0.07 ± 0.04	0.07 ± 0.04
Right Cochlea	162 out of 162	0.09 ± 0.04	0.09 ± 0.04
Oesophagus	110 out of 154	0.30 ± 0.40	0.14 ± 0.06
Left Eye	160 out of 162	0.08 ± 0.04	0.08 ± 0.03
Right Eye	161 out of 162	0.08 ± 0.04	0.07 ± 0.03
Left Lacrimal Gland	161 out of 162	0.07 ± 0.04	0.06 ± 0.03
Right Lacrimal Gland	161 out of 162	0.06 ± 0.04	0.06 ± 0.03
Left Submandibular Gland	78 out of 150	0.45 ± 0.40	0.16 ± 0.06
Right Submandibular Gland	84 out of 156	0.39 ± 0.30	0.18 ± 0.06
Thyroid Gland	94 out of 153	0.56 ± 0.68	0.16 ± 0.07
Larynx	110 out of 138	0.28 ± 0.29	0.17 ± 0.06
Left Lens	145 out of 146	0.12 ± 0.06	0.12 ± 0.06
Right Lens	133 out of 133	0.11 ± 0.05	0.11 ± 0.05
Lips	156 out of 162	0.13 ± 0.07	0.12 ± 0.05
Constrictor Muscle	150 out of 162	0.18 ± 0.27	0.12 ± 0.05
Left Sternocleidomastoid Muscle	134 out of 162	0.36 ± 0.61	0.15 ± 0.07

Right Sternocleidomastoid Muscle	125 out of 162	0.28 ± 0.39	0.14 ± 0.06
Optic Chiasm	161 out of 162	0.12 ± 0.06	0.12 ± 0.05
Left Optic Nerve	159 out of 161	0.10 ± 0.05	0.10 ± 0.05
Right Optic Nerve	156 out of 162	0.11 ± 0.07	0.10 ± 0.05
Left Parotid	155 out of 162	0.14 ± 0.08	0.13 ± 0.05
Right Parotid	153 out of 162	0.15 ± 0.09	0.14 ± 0.06
Pituitary	162 out of 162	0.08 ± 0.03	0.08 ± 0.03
Spinal Cord	50 out of 154	1.36 ± 1.39	0.13 ± 0.09
Anterior Aorta Base	129 out of 162	0.21 ± 0.15	0.15 ± 0.07
Left Anterior Descending Artery	80 out of 142	0.47 ± 0.53	0.18 ± 0.06
Left Atrium	131 out of 150	0.19 ± 0.10	0.16 ± 0.06
Right Atrium	124 out of 150	0.22 ± 0.13	0.17 ± 0.06
Left Brachial Plexus	59 out of 84	0.29 ± 0.25	0.15 ± 0.07
Right Brachial Plexus	58 out of 84	0.26 ± 0.24	0.12 ± 0.05
Bronchus	142 out of 162	0.15 ± 0.10	0.12 ± 0.05
Cardiac Avoidance Area	125 out of 150	0.21 ± 0.11	0.17 ± 0.05
Left Chestwall	144 out of 162	0.15 ± 0.14	0.11 ± 0.06
Right Chestwall	148 out of 162	0.14 ± 0.10	0.11 ± 0.05
Heart	106 out of 144	0.23 ± 0.12	0.17 ± 0.06
Heart and Anterior Pulmonary Artery	112 out of 144	0.21 ± 0.11	0.16 ± 0.06
Liver	76 out of 132	0.40 ± 0.44	0.16 ± 0.07
Left Lung	80 out of 96	0.17 ± 0.22	0.08 ± 0.05
Right Lung	110 out of 114	0.09 ± 0.07	0.08 ± 0.05
Ribs	145 out of 162	0.14 ± 0.12	0.11 ± 0.07
Spinal Cord	108 out of 144	0.19 ± 0.19	0.09 ± 0.05
Inferior Vena Cava	91 out of 144	0.33 ± 0.27	0.18 ± 0.06
Superior Vena Cava	145 out of 162	0.16 ± 0.10	0.13 ± 0.06
Left Ventricle	117 out of 144	0.23 ± 0.12	0.18 ± 0.06
Right Ventricle	105 out of 144	0.26 ± 0.12	0.19 ± 0.05
Anterior Aorta	45 out of 54	0.17 ± 0.12	0.13 ± 0.08

Oesophagus	23 out of 24	0.15 ± 0.06	0.14 ± 0.05
Great Vessels	48 out of 54	0.17 ± 0.10	0.15 ± 0.07
Left Lung	12 out of 12	0.12 ± 0.10	0.12 ± 0.10
Stomach	22 out of 78	0.81 ± 0.75	0.12 ± 0.06
Trachea	20 out of 24	0.15 ± 0.10	0.11 ± 0.05
Left Humerus	4 out of 6	0.23 ± 0.21	0.11 ± 0.01
Right Humerus	6 out of 6	0.19 ± 0.04	0.19 ± 0.04
Trachea	77 out of 78	0.12 ± 0.07	0.11 ± 0.07
Anterior Aorta	50 out of 54	0.13 ± 0.08	0.12 ± 0.06
Pulmonary Artery	40 out of 42	0.11 ± 0.07	0.10 ± 0.05
Oesophagus	53 out of 66	0.54 ± 1.06	0.14 ± 0.05
Great Vessels	54 out of 54	0.13 ± 0.06	0.13 ± 0.06
Lungs	36 out of 36	0.07 ± 0.05	0.07 ± 0.05
Atrioventricular Node	36 out of 42	0.20 ± 0.15	0.15 ± 0.06
Pericardium	27 out of 42	0.23 ± 0.14	0.15 ± 0.07
Spleen	12 out of 12	0.13 ± 0.07	0.13 ± 0.07
Liver	5 out of 6	0.28 ± 0.11	0.24 ± 0.06
Heart	6 out of 6	0.14 ± 0.01	0.14 ± 0.01
Heart and Anterior Pulmonary Artery	6 out of 6	0.08 ± 0.01	0.08 ± 0.01
Right Lung	6 out of 6	0.04 ± 0.00	0.04 ± 0.00
Left Humerus	12 out of 18	0.27 ± 0.32	0.05 ± 0.04
Right Humerus	11 out of 12	0.09 ± 0.11	0.06 ± 0.05
Stomach	7 out of 30	0.57 ± 0.33	0.20 ± 0.05
Left Brachial Plexus	10 out of 18	0.29 ± 0.16	0.17 ± 0.09
Right Brachial Plexus	6 out of 13	0.79 ± 0.68	0.09 ± 0.01
Spleen	6 out of 6	0.13 ± 0.08	0.13 ± 0.08
Anterior-Inferior Aorta	4 out of 8	0.34 ± 0.25	0.12 ± 0.09
Inferior Vena Cava	0 out of 6	1.04 ± 0.14	N/A

Table S5: A summary of the DVH metric uncertainties for all OARs in our 2 patient cohorts using 16 registrations, including the count of OARs that passed our set mDTA threshold. The statistics are for the OARs whose registrations were plausible. Please note that the total count of the OARs considered varied in some cases, if the OAR was within the target volume or due to failure in the auto-segmentation. Some OAR names are duplicates due to instances of partial OAR being clinically delineated. These results were not merged with whole organ results, such as the stomach in the lung cohort.

**OAR**	**Number of Plausible Registrations out of the total count of OARs considered**	**Mean ± Standard Deviation of mDTA (cm)**	**Mean ± Standard Deviation of mDTA (cm) for Plausible Registrations**
Mandible	432 out of 432	0.05 ± 0.03	0.05 ± 0.03
Left Brachial Plexus	393 out of 416	0.17 ± 0.16	0.14 ± 0.05
Right Brachial Plexus	362 out of 416	0.18 ± 0.18	0.13 ± 0.04
Brain	432 out of 432	0.04 ± 0.02	0.04 ± 0.02
Brainstem	431 out of 432	0.11 ± 0.04	0.11 ± 0.04
Oral Cavity	394 out of 432	0.19 ± 0.08	0.17 ± 0.06
Left Cochlea	432 out of 432	0.07 ± 0.04	0.07 ± 0.04
Right Cochlea	431 out of 431	0.09 ± 0.04	0.09 ± 0.04
Oesophagus	321 out of 416	0.27 ± 0.39	0.14 ± 0.06
Left Eye	432 out of 432	0.07 ± 0.02	0.07 ± 0.02
Right Eye	432 out of 432	0.07 ± 0.02	0.07 ± 0.02
Left Lacrimal Gland	432 out of 432	0.06 ± 0.03	0.06 ± 0.03
Right Lacrimal Gland	432 out of 432	0.06 ± 0.02	0.06 ± 0.02
Left Submandibular Gland	224 out of 399	0.43 ± 0.40	0.15 ± 0.06
Right Submandibular Gland	241 out of 416	0.37 ± 0.29	0.17 ± 0.06
Thyroid Gland	269 out of 416	0.54 ± 0.68	0.16 ± 0.07
Larynx	309 out of 370	0.26 ± 0.29	0.17 ± 0.06
Left Lens	392 out of 400	0.12 ± 0.06	0.11 ± 0.06
Right Lens	362 out of 365	0.11 ± 0.05	0.11 ± 0.05
Lips	427 out of 432	0.12 ± 0.06	0.12 ± 0.05
Constrictor Muscle	409 out of 432	0.16 ± 0.22	0.12 ± 0.05
Left Sternocleidomastoid Muscle	380 out of 432	0.33 ± 0.59	0.14 ± 0.06
Right Sternocleidomastoid Muscle	334 out of 432	0.27 ± 0.38	0.13 ± 0.05
Optic Chiasm	429 out of 432	0.12 ± 0.06	0.12 ± 0.05

Left Optic Nerve	431 out of 432	0.10 ± 0.05	0.10 ± 0.05
Right Optic Nerve	419 out of 432	0.11 ± 0.06	0.10 ± 0.05
Left Parotid	417 out of 432	0.13 ± 0.07	0.12 ± 0.05
Right Parotid	412 out of 432	0.14 ± 0.08	0.13 ± 0.06
Pituitary	432 out of 432	0.08 ± 0.03	0.08 ± 0.03
Spinal Cord	149 out of 416	1.36 ± 1.43	0.12 ± 0.09
Anterior Aorta Base	388 out of 432	0.17 ± 0.10	0.14 ± 0.07
Left Anterior Descending Artery	234 out of 382	0.47 ± 0.57	0.18 ± 0.06
Left Atrium	375 out of 400	0.16 ± 0.07	0.15 ± 0.05
Right Atrium	362 out of 400	0.18 ± 0.11	0.16 ± 0.05
Left Brachial Plexus	153 out of 224	0.29 ± 0.25	0.14 ± 0.06
Right Brachial Plexus	161 out of 224	0.24 ± 0.22	0.12 ± 0.06
Bronchus	389 out of 432	0.14 ± 0.08	0.11 ± 0.04
Cardiac Avoidance Area	371 out of 400	0.18 ± 0.09	0.16 ± 0.05
Left Chestwall	384 out of 432	0.16 ± 0.15	0.11 ± 0.06
Right Chestwall	391 out of 432	0.14 ± 0.10	0.11 ± 0.05
Heart	294 out of 384	0.21 ± 0.10	0.16 ± 0.06
Heart and Anterior Pulmonary Artery	313 out of 384	0.19 ± 0.10	0.16 ± 0.05
Liver	235 out of 352	0.35 ± 0.41	0.15 ± 0.07
Left Lung	218 out of 256	0.15 ± 0.18	0.08 ± 0.05
Right Lung	296 out of 304	0.08 ± 0.06	0.07 ± 0.04
Ribs	387 out of 432	0.14 ± 0.12	0.10 ± 0.07
Spinal Cord	304 out of 384	0.17 ± 0.18	0.09 ± 0.05
Inferior Vena Cava	275 out of 384	0.30 ± 0.26	0.17 ± 0.06
Superior Vena Cava	409 out of 432	0.14 ± 0.08	0.13 ± 0.06
Left Ventricle	336 out of 384	0.20 ± 0.10	0.17 ± 0.06
Right Ventricle	322 out of 384	0.23 ± 0.10	0.19 ± 0.05
Anterior Aorta	124 out of 144	0.15 ± 0.11	0.12 ± 0.08
Oesophagus	64 out of 64	0.13 ± 0.04	0.13 ± 0.04
Great Vessels	134 out of 144	0.16 ± 0.09	0.14 ± 0.07
Left Lung	32 out of 32	0.10 ± 0.09	0.10 ± 0.09

Stomach	61 out of 208	0.81 ± 0.79	0.12 ± 0.06
Trachea	57 out of 64	0.14 ± 0.09	0.11 ± 0.06
Left Humerus	16 out of 16	0.12 ± 0.02	0.12 ± 0.02
Right Humerus	16 out of 16	0.17 ± 0.02	0.17 ± 0.02
Trachea	207 out of 208	0.11 ± 0.07	0.11 ± 0.07
Anterior Aorta	136 out of 144	0.11 ± 0.07	0.10 ± 0.06
Pulmonary Artery	112 out of 112	0.08 ± 0.02	0.08 ± 0.02
Oesophagus	144 out of 176	0.52 ± 1.05	0.13 ± 0.05
Great Vessels	144 out of 144	0.11 ± 0.05	0.11 ± 0.05
Lungs	96 out of 96	0.06 ± 0.03	0.06 ± 0.03
Atrioventricular Node	110 out of 112	0.15 ± 0.06	0.15 ± 0.06
Pericardium	75 out of 112	0.21 ± 0.12	0.14 ± 0.07
Spleen	32 out of 32	0.13 ± 0.07	0.13 ± 0.07
Liver	16 out of 16	0.22 ± 0.06	0.22 ± 0.06
Heart	16 out of 16	0.14 ± 0.01	0.14 ± 0.01
Heart and Anterior Pulmonary Artery	16 out of 16	0.08 ± 0.01	0.08 ± 0.01
Right Lung	16 out of 16	0.04 ± 0.01	0.04 ± 0.01
Left Humerus	32 out of 48	0.25 ± 0.30	0.04 ± 0.03
Right Humerus	32 out of 32	0.04 ± 0.01	0.04 ± 0.01
Stomach	17 out of 80	0.52 ± 0.30	0.19 ± 0.04
Left Brachial Plexus	24 out of 48	0.30 ± 0.15	0.16 ± 0.07
Right Brachial Plexus	16 out of 35	0.79 ± 0.65	0.09 ± 0.01
Spleen	16 out of 16	0.10 ± 0.05	0.10 ± 0.05
Anterior-Inferior Aorta	15 out of 21	0.30 ± 0.25	0.16 ± 0.09
Inferior Vena Cava	0 out of 16	1.10 ± 0.15	N/A

Comparing Nested Registrations (4 vs 16)

While our work sought to increase the number of registrations to check whether a pragmatic approach was appropriate and could offer dose mapping uncertainty estimation, we dropped one parameter in the 6 registrations due to computational resource constraints.

When we compared four registrations from the set that had six registrations, with the 16 registrations that were an extension of the four, we observed definitively that the geometric and dosimetric trends between the two sets of registrations were similar. The lower performance in the set with six registrations was due to the generally poor performance of registrations when only one resolution level was used. Here we compare the results, excluding the one resolution level which may bias the results.

Figure S5: Plausible registrations across all registrations and structures discriminated by patient. Interestingly, the pattern of plausible registrations for 4 and 16 registrations is very similar, evidencing the bias introduced by including resolution level 1.

**Head and Neck Cancer cohort**	**Lung Cancer Cohort**

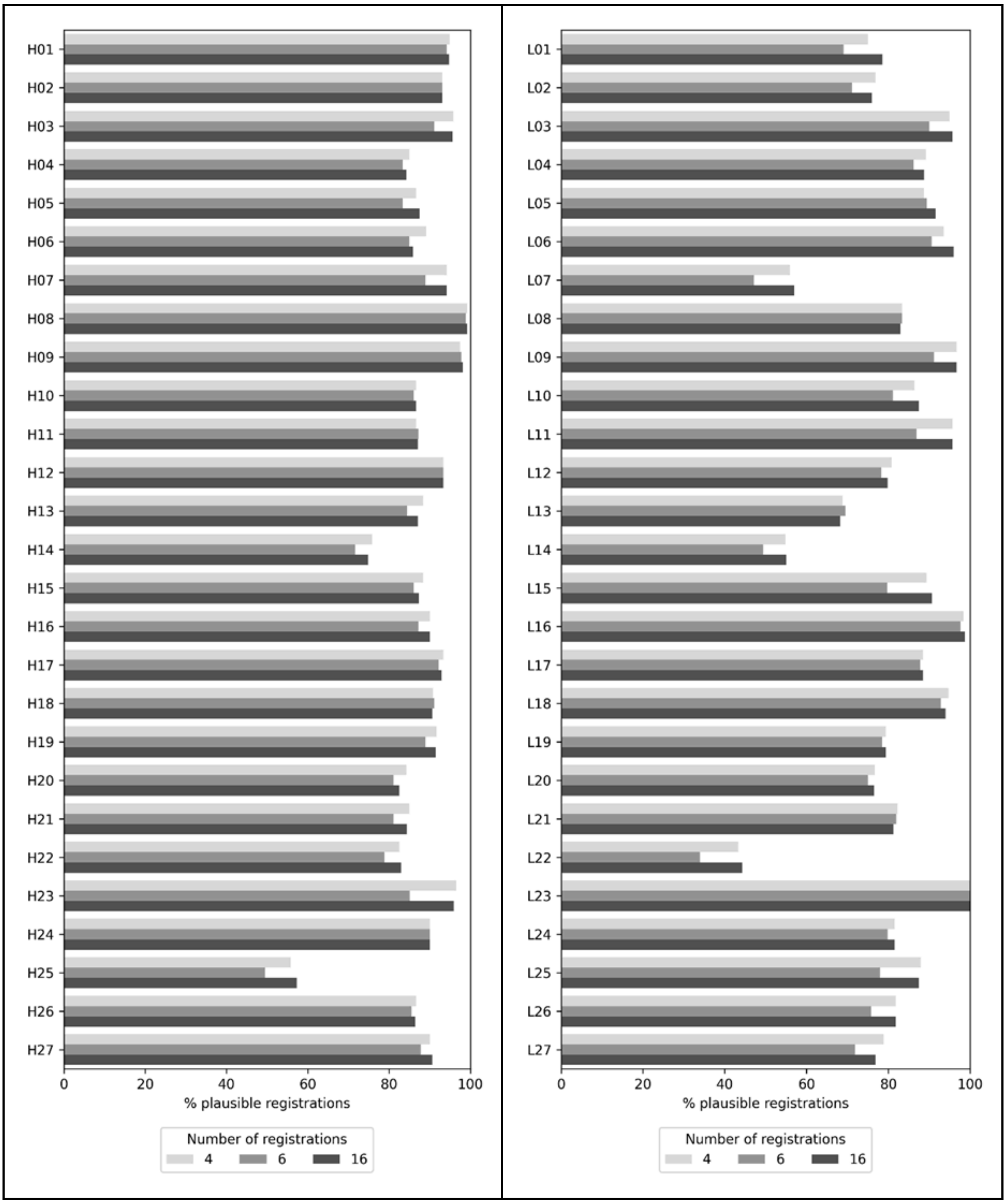

Figure S6: Plausible registrations across all patients and registrations discriminated by structure name for **the head and neck cancer cohort.** Similarly, the pattern of plausible registrations for 4 and 16 registrations is very similar, evidencing the bias introduced by including resolution level 1.

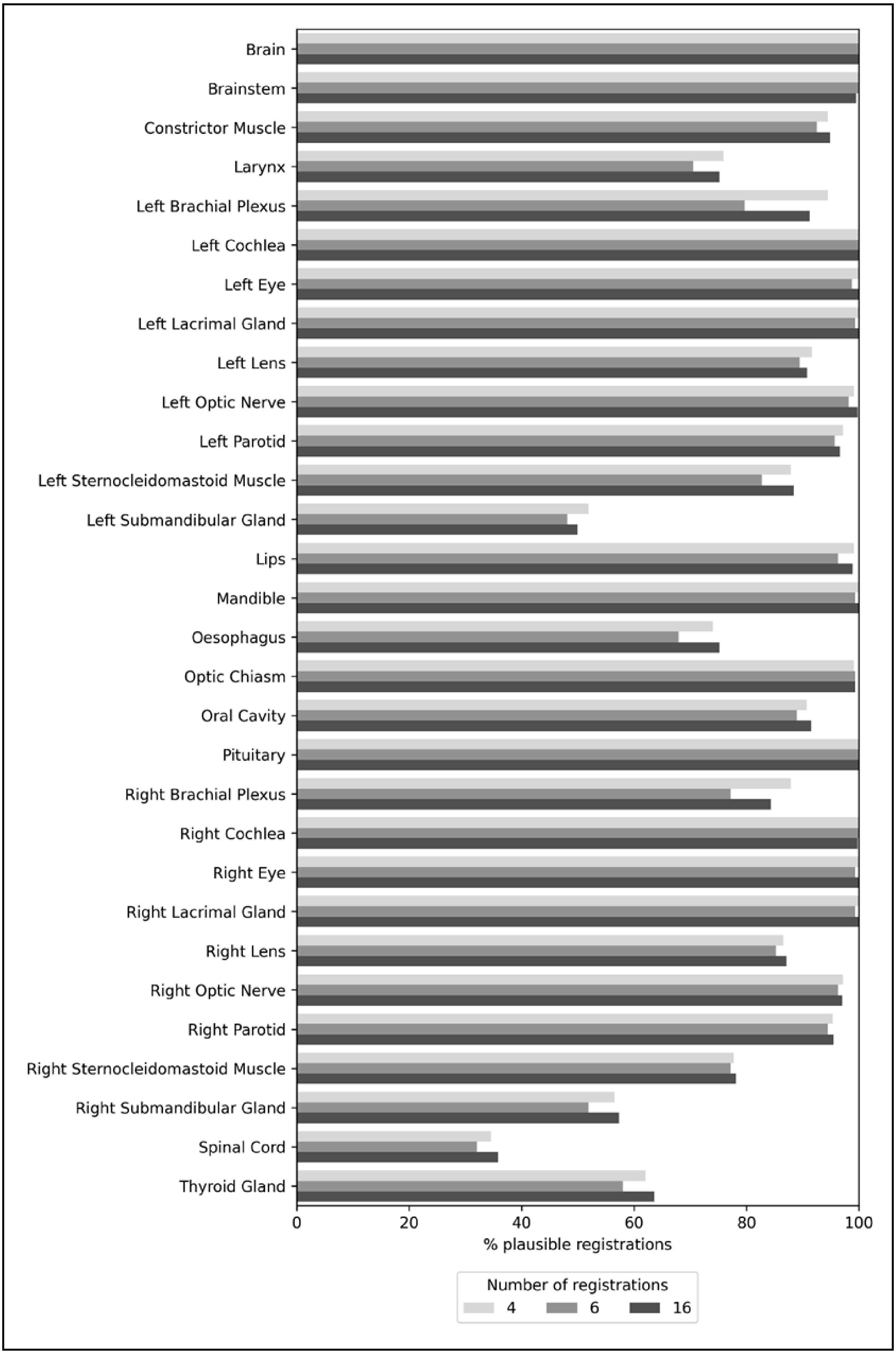
Brain
Brainstem
Constrictor Muscle
Larynx
Left Brachial Plexus
Left Cochlea
Left Eye
Left Lacrimal Gland
Left Lens
Left Optic Nerve
Left Parotid
Left Sternocleidomastoid Muscle
Left Submandibular Gland
Lips
Mandible
Oesophagus
Optic Chiasm
Oral Cavity
Pituitary
Right Brachial Plexus
Right Cochlea
Right Eye
Right Lacrimal Gland
Right Lens
Right Optic Nerve
Right Parotid
Right Sternocleidomastoid Muscle
Right Submandibular Gland
Spinal Cord
Thyroid Gland
0
20
40
60
80
100
% plausible registrations
Number of registrations
4
6
16

Figure S7: Plausible registrations across all patients and registrations discriminated by structure name for the **lung cancer cohort**. Similarly, the pattern of plausible registrations for 4 and 16 registrations is very similar, evidencing the bias introduced by including resolution level 1.

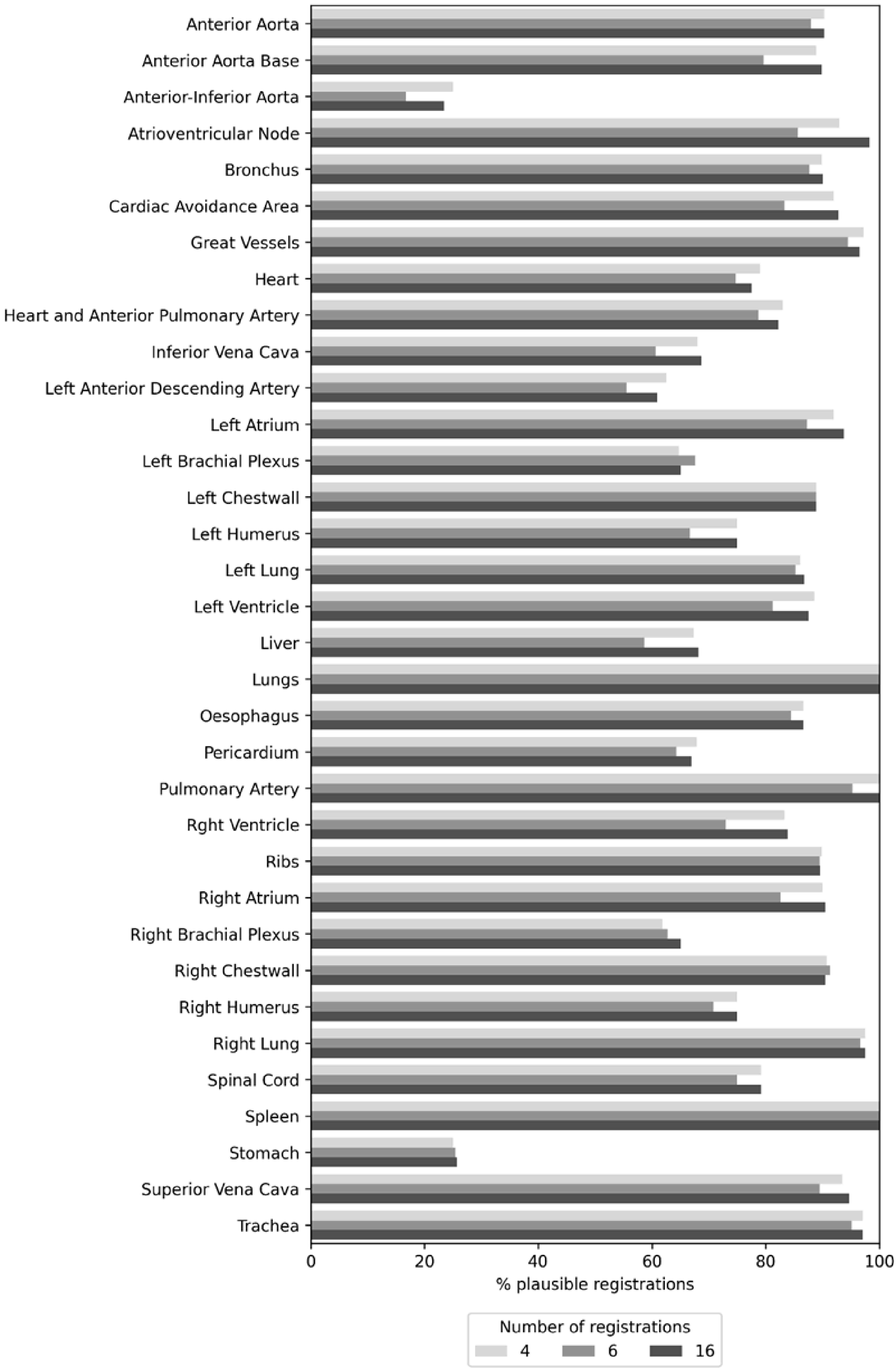
Anterior Aorta
Anterior Aorta Base
Anterior-Inferior Aorta
Atrioventricular Node
Bronchus
Cardiac Avoidance Area
Great Vessels
Heart
Heart and Anterior Pulmonary Artery
Inferior Vena Cava
Left Anterior Descending Artery
Left Atrium
Left Brachial Plexus
Left Chestwall
Left Humerus
Left Lung
Left Ventricle
Liver
Lungs
Oesophagus
Pericardium
Pulmonary Artery
Rght Ventricle
Ribs
Right Atrium
Right Brachial Plexus
Right Chestwall
Right Humerus
Right Lung
Spinal Cord
Spleen
Stomach
Superior Vena Cava
Trachea
0
20
40
60
80
100
% plausible registrations
Number of registrations
4
6
16

Figure S8: Plausible registrations across all patients and structures discriminated by registration name. Common registrations are marked by a star. The registration name indicates: metric, resolution levels, grid weight and initial Gaussian blurring

**Head and Neck Cancer cohort**	**Lung Cancer Cohort**

6 registrations

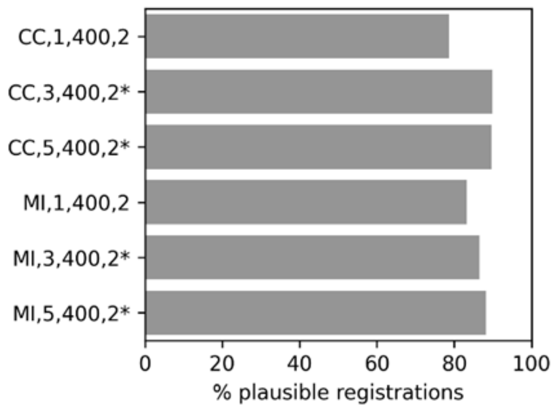

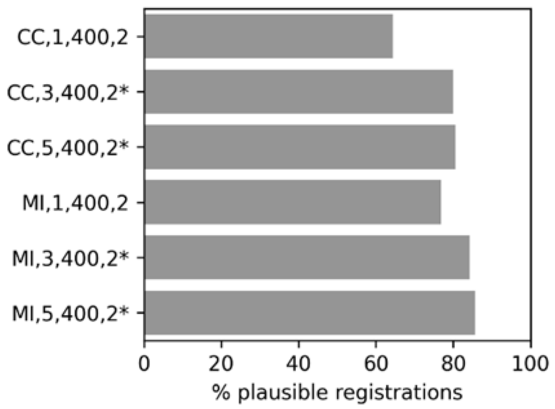

16 registrations

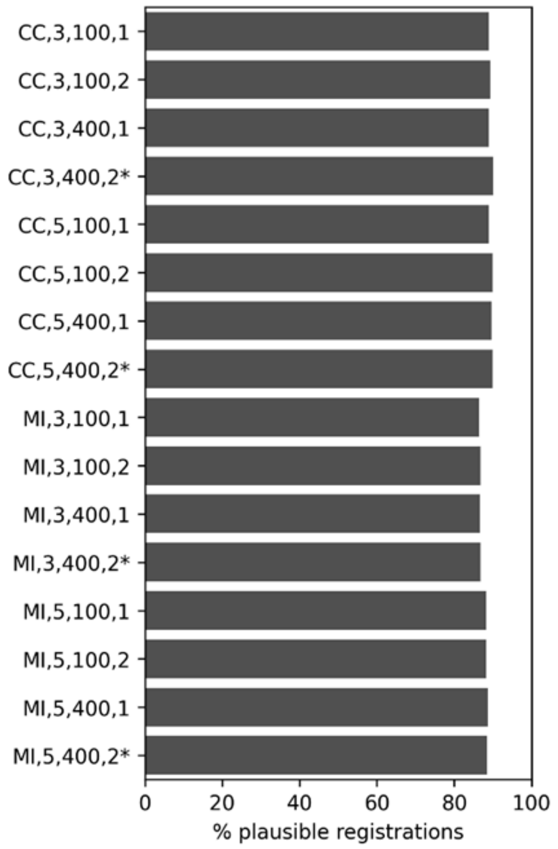

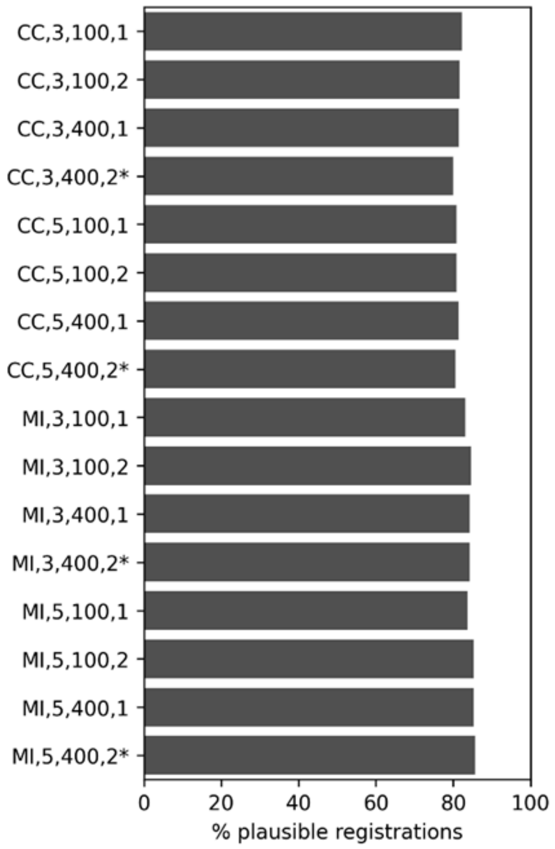

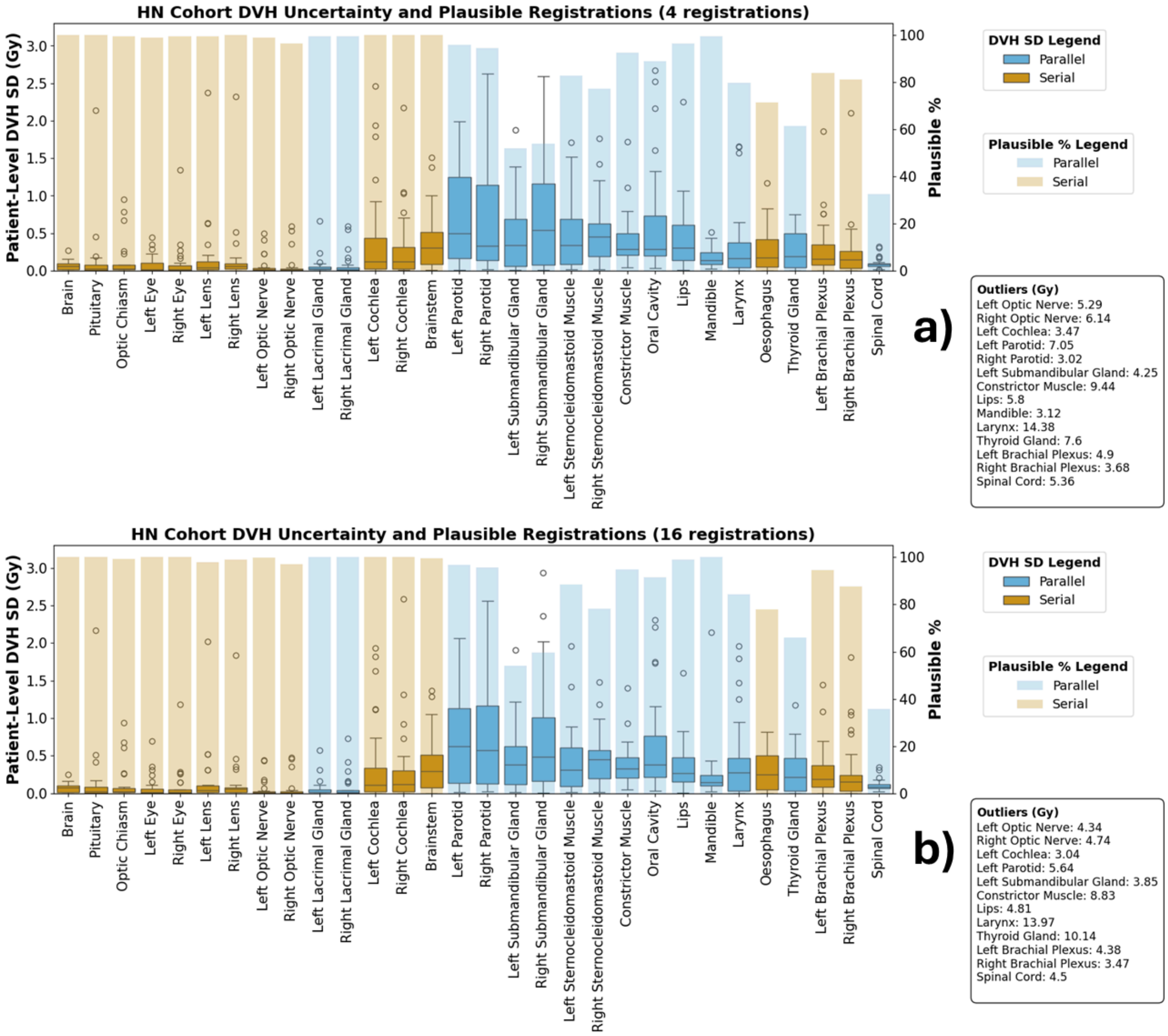

Figure S9: A summary of the DVH metric uncertainties for all OARs in the head and neck cohort using 4 (a) and 16 (b) registrations, organised by the general cranial-caudal format. For readability, outliers of each box plot are listed below the legends. The bar plots show the percentage of OARs that passed our set mDTA threshold of 0.3 cm (y-axis on the right-hand side of the plot). The box plots show the standard deviation of the mean dose for the parallel OARs and D0.1 cm^3 for the serial OARs. **Similar** trends in performance are observed between the two sets of registrations

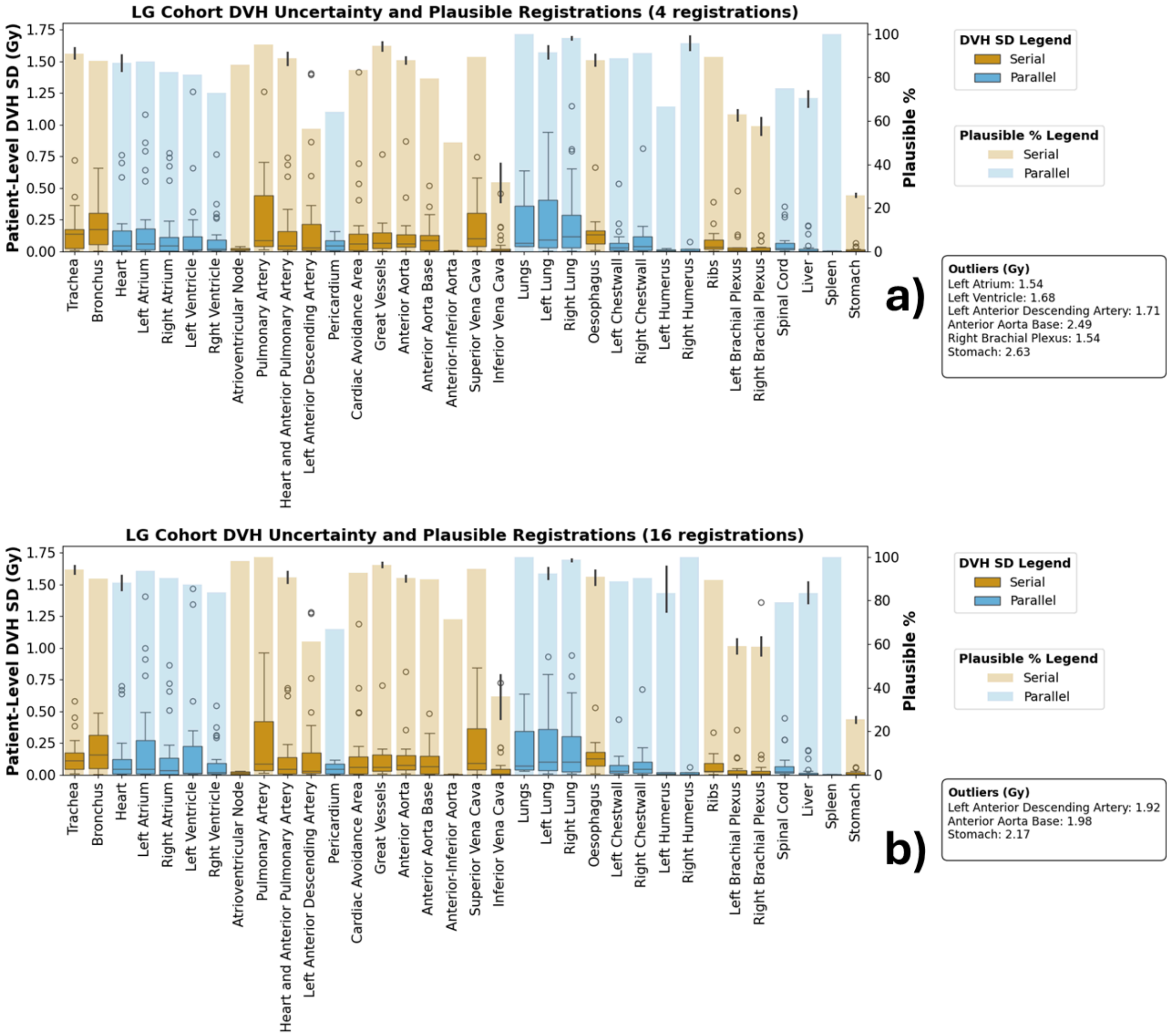

Figure S10: A summary of the DVH metric uncertainties for all OARs in the lung cohort using 4 (a) and 16 (b) registrations, organised by the general cranial-caudal format. For readability, outliers of each box plot are listed below the legends. The bar plots show the percentage of OARs that passed our set mDTA threshold of 0.3 cm (y-axis on the right-hand side of the plot). The box plots show the standard deviation of the mean dose for the parallel OARs and D0.1 cm^3 for the serial OARs. **Similar** trends in performance are observed between the two sets of registrations.

DIR using open-source research tools

On 10 arbitrary patients, 5 from each cohort, we ran the image registration part of our pipeline using an open-source research toolkit (NiftyReg). The algorithm settings were as shown in Figure S11. From Figure S12, we see that NiftyReg registrations generally resulted in larger meanDTA values compared to RayStation for our patient cohort. This means that NiftyReg performed poorly compared to ANACONDA-based (from RayStation) registrations, e.g., 84% of registrations passed the 0.3 cm threshold for ANACONDA, but 6% passed for NiftyReg. As a caveat, these open-source tools are not fine-tuned for intra-patient registration in the radiotherapy context, in contrast to commercially available tools used for clinical applications. As the results were clearly poorer, we did not estimate dose mapping uncertainties.

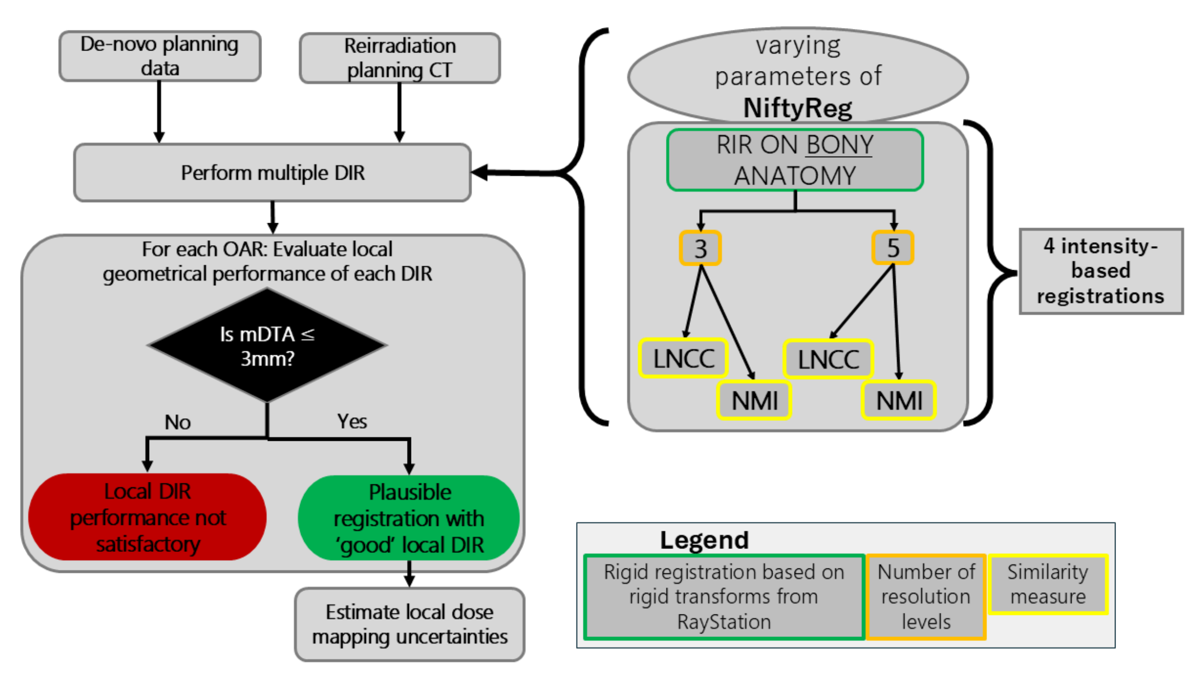

Figure S11: Flowchart of the script for our open-source pipeline that estimates dose mapping uncertainty using **four** registrations.
Abbreviations: LNCC - correlation coefficient; NMI - mutual information.

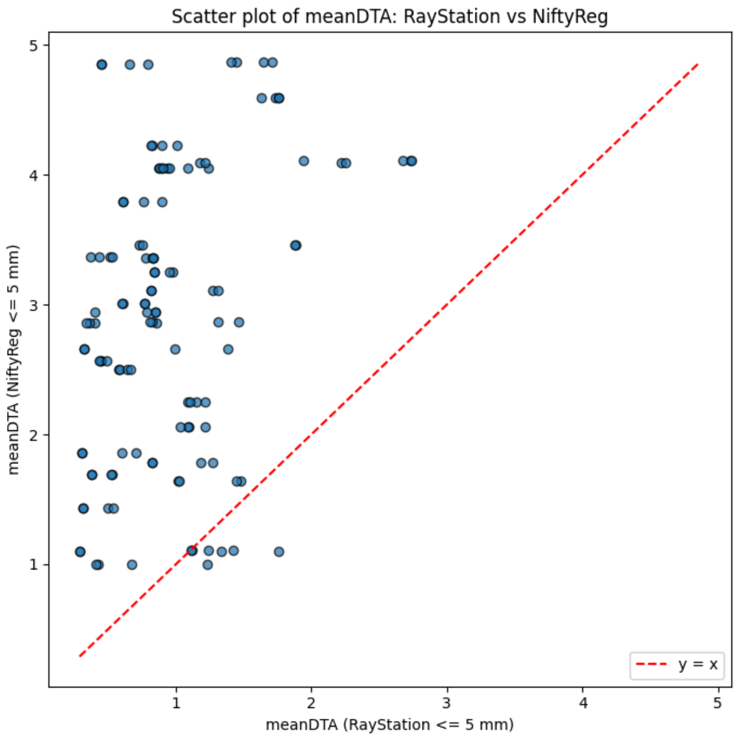

Figure S12: A scatter plot comparing the geometric performance of both algorithms, ANACONDA from RayStation and NiftyReg (Python-based) and varying the similarity measure in the 2 algorithms (correlation coefficient, and mutual information) and 2 numbers of resolution levels (3 and 5) NiftyReg generally resulted in larger meanDTA values for our patient cohort compared to RayStation. The plot has been truncated to 0.5 cm for readability

References

[1] Weistrand O, Svensson S. The ANACONDA algorithm for deformable image registration in radiotherapy. Medical Physics 2015;42. https://doi.org/10.1118/1.4894702.

[2] Rigaud B, Simon A, Castelli J, Gobeli M, Ospina Arango J-D, Cazoulat G, et al. Evaluation of Deformable Image Registration Methods for Dose Monitoring in Head and Neck Radiotherapy. BioMed Research International 2015;2015:726268. https://doi.org/10.1155/2015/726268.

[3] Ratke A, Darsht E, Heinzelmann F, Kröninger K, Timmermann B, Bäumer C. Deep-learning-based deformable image registration of head CT and MRI scans. Front Phys 2023;11. https://doi.org/10.3389/fphy.2023.1292437.

[4] Brock KK, Mutic S, McNutt TR, Li H, Kessler ML. Use of image registration and fusion algorithms and techniques in radiotherapy: Report of the AAPM Radiation Therapy Committee Task Group No. 132: Report. Medical Physics 2017;44. https://doi.org/10.1002/mp.12256.

[5] Nenoff L, Amstutz F, Murr M, Archibald-Heeren B, Fusella M, Hussein M, et al. Review and recommendations on deformable image registration uncertainties for radiotherapy applications. Physics in Medicine & Biology 2023;68:24TR01. https://doi.org/10.1088/1361-6560/AD0D8A.

[6] Staring M, Klein S, Pluim JPW. Nonrigid registration with tissue-dependent filtering of the deformation field. Phys Med Biol 2007;52:6879. https://doi.org/10.1088/0031-9155/52/23/007.

[7] Polo AL, Nix M, Thompson C, O'Hara C, Entwisle J, Murray L, et al. Improving hybrid image and structure-based deformable image registration for large internal deformations. Phys Med Biol 2024;69:095011. https://doi.org/10.1088/1361-6560/ad3723.

[8] Johnston SM, Johnson GA, Badea CT. Temporal and spectral imaging with micro-CT. Medical Physics 2012;39:4943–58. https://doi.org/10.1118/1.4736809.
